# Synthetic Cannabinoid (Mojo)-Induced ST-Segment Elevation Myocardial Infarction: A Case Report

**DOI:** 10.7759/cureus.84570

**Published:** 2025-05-21

**Authors:** Kubiat E Udoh, Kuseme E Udoh, Andikan E Udoh

**Affiliations:** 1 Internal Medicine, Baton Rouge General, Baton Rouge, USA

**Keywords:** cannabis, st-segment elevation myocardial infarction (stemi), synthetic cannabinoids, synthetic marijuana, substance abuse

## Abstract

Synthetic cannabinoids, such as Mojo, are increasingly used among adolescents and young adults due to their perceived legality and accessibility. These compounds exhibit significantly greater potency than natural cannabis, with full agonist activity at cannabinoid receptors, and have been associated with a range of adverse effects, including cardiovascular toxicity. The underlying mechanisms may involve sympathetic stimulation, platelet aggregation, and endothelial injury, which can precipitate acute coronary events even in otherwise healthy individuals.

We report the case of a 32-year-old man with no significant medical history and an atherosclerotic cardiovascular disease (ASCVD) risk score of 3.6%, who presented with acute chest pain four hours after smoking the synthetic cannabinoid Mojo. Electrocardiogram revealed ST-segment elevations, and emergent left heart catheterization demonstrated acute thrombotic total occlusion of the left circumflex artery. The patient was successfully treated with percutaneous coronary intervention (PCI) and stent placement. Urine drug screening confirmed recent synthetic cannabinoid use. He had no identifiable traditional cardiovascular risk factors or family history of heart disease.

This case highlights synthetic cannabinoids as an emerging and underrecognized cause of acute coronary syndrome in young adults. Clinicians should maintain a high index of suspicion for synthetic cannabinoid use in young patients presenting with myocardial infarction without conventional risk factors. A simple urine drug screen can be a critical diagnostic tool. This report emphasizes the need for increased awareness, targeted patient education, and further research into the cardiovascular risks posed by these substances.

## Introduction

Cannabis is one of the most widely used psychoactive substances globally, with particularly high prevalence among adolescents and young adults. This trend is driven by increasing legalization and growing social acceptance in the United States and many parts of the world [1,2]. While natural cannabis has been extensively studied, synthetic cannabinoids represent a newer class of compounds that mimic the effects of tetrahydrocannabinol (THC), the primary psychoactive constituent of cannabis, but often with far greater potency and unpredictable physiological consequences [2-4].

Mojo is a synthetic cannabinoid that exemplifies this risk. Unlike THC, which acts as a partial agonist at cannabinoid receptors, Mojo binds with high affinity and acts as a full agonist at both CB1 and CB2 receptors [2-5]. This enhanced receptor activity may explain its more intense and sometimes toxic effects, particularly on the cardiovascular system.

The use of synthetic cannabinoids poses a growing public health concern, particularly among young individuals who may perceive these substances as safe or legal alternatives to cannabis. This case highlights the potential for synthetic cannabinoids to precipitate acute coronary events, even in individuals with minimal traditional cardiovascular risk.

We report the case of a 32-year-old man with a low atherosclerotic cardiovascular disease (ASCVD) risk score of 3.6%, who presented with an ST-segment elevation myocardial infarction (STEMI) just hours after using Mojo. This case underscores the need for heightened clinical awareness, public education, and further research into the cardiovascular risks of synthetic cannabinoids.

## Case presentation

A 32-year-old African-American man with no medical history presented to the hospital with complaints of chest pain, which began within four hours after Mojo use. He denied any personal or family history of heart disease but admitted to frequent synthetic cannabinoid use and daily alcohol use.

**Figure 1:**
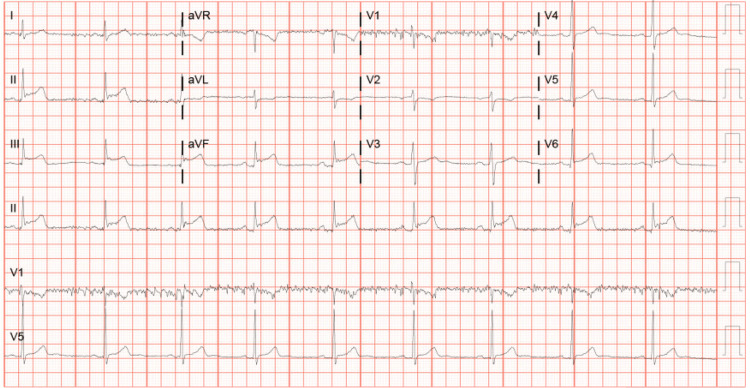
EKG showing ST-segment elevations in leads II, III, and aVF and T-wave inversions in aVL EKG: electrocardiogram

**Figure 2:**
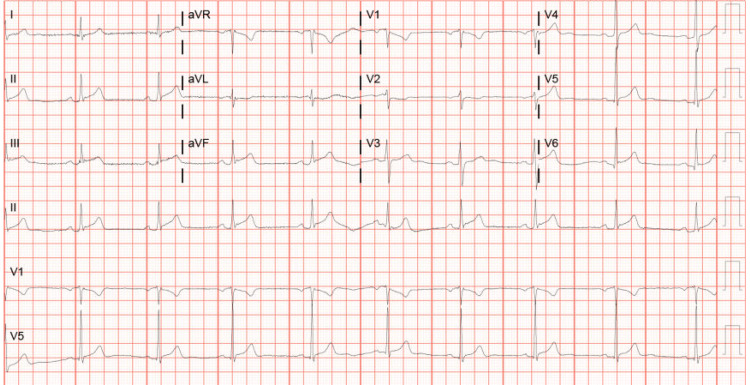
Serial EKG showing progressive changes in leads II, III, and aVF EKG: electrocardiogram

**Figure 3:**
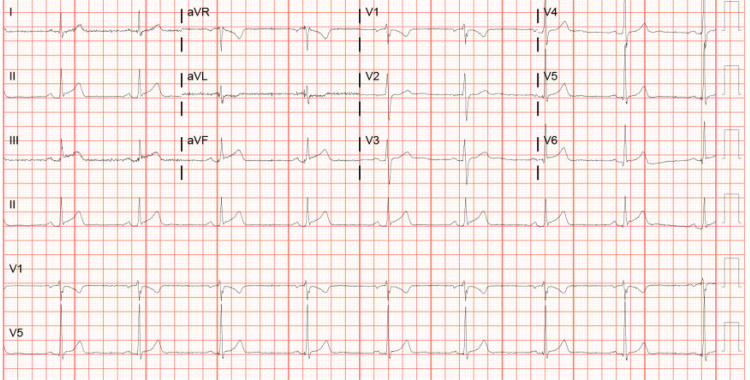
Serial EKG showing progressive changes in leads II, III, and aVF EKG: electrocardiogram

On arrival at the emergency room, his vital signs were stable: blood pressure 132/81 mmHg, pulse 55 beats per minute, oxygen saturation 98% on room air, and temperature 98.2°F. Moreover, his physical exam was unremarkable. An electrocardiogram (EKG) that showed ST-segment elevations in leads II, III, and aVF and T-wave inversions in aVL was immediately obtained. He received aspirin 321 mg and IV morphine 4 mg and reported complete resolution of his symptoms. Laboratory results were significant for troponin levels of 1071.8 pg/ml with a peak of 249,823 pg/ml, hemoglobin A1c 5.5%, and thyroid-stimulating hormone 0.982 uIU/mL. A urine drug screen was positive for cannabinoids. Serial EKGs are illustrated below (Figures 1-3).

**Figure 4:**
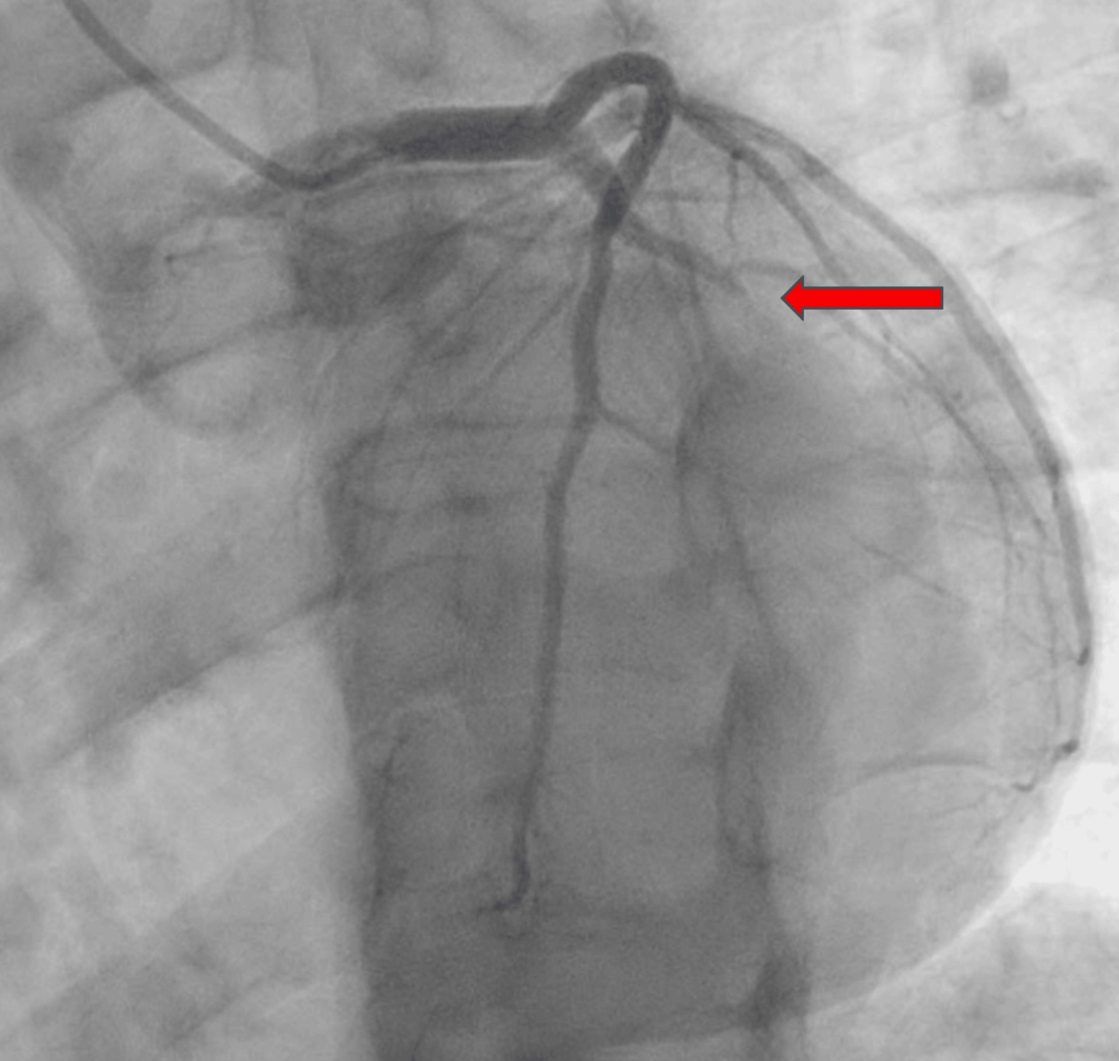
LAO caudal view pre-PCI showing total occlusion to the mid-left circumflex artery (red arrow) LAO: left anterior oblique; PCI: percutaneous coronary intervention

**Figure 5:**
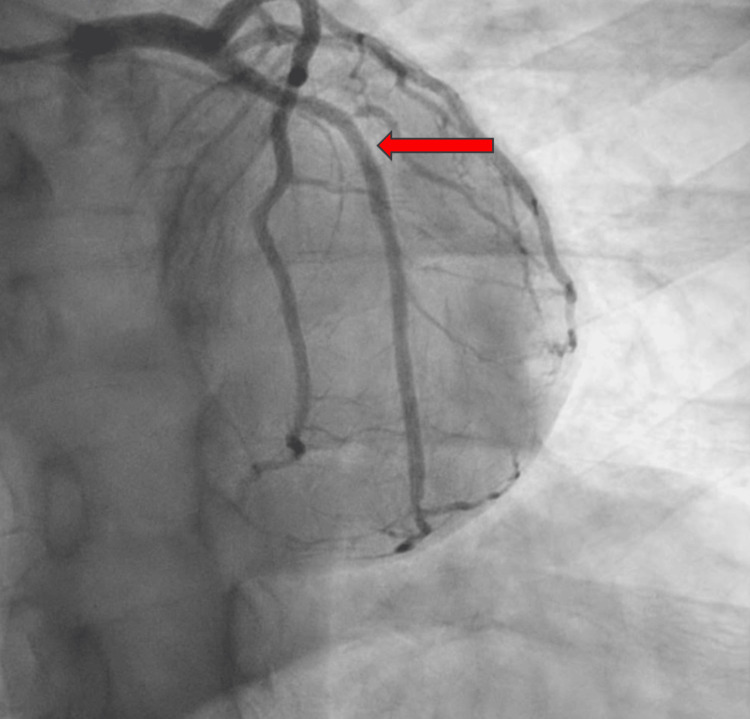
LAO caudal view post-PCI showing the mid-left circumflex artery after the deployment of a 3×20 mm drug-eluting stent (red arrow) LAO: left anterior oblique; PCI: percutaneous coronary intervention

2D echocardiogram revealed normal left ventricular cavity size with low-normal left ventricular systolic function, left ventricular ejection fraction (LVEF) 50-55%, and moderate asymmetrical hypertrophy. He underwent an emergent left heart coronary angiography, which revealed an acute thrombotic total occlusion to the mid-left circumflex artery (LCX); the choice of therapy was percutaneous coronary intervention (PCI) with the deployment of a 3×20 mm drug-eluting stent. Left heart catheterization (LHC) illustrates the occluded LCX pre- and post-PCI (Figures 4-5).

He was initiated on routine post-PCI therapy: aspirin and ticagrelor for dual antiplatelet therapy, atorvastatin for plaque stabilization, and metoprolol for rate control. He remained asymptomatic and was discharged home after extensive counselling on the dangers of recreational drugs, alcohol, and smoking. Follow-up at the cardiology clinic was set up, and resources regarding substance abuse were provided to the patient prior to discharge.

## Discussion

Mojo is a synthetic cannabinoid that has become increasingly popular among teenagers and young adults. Based on the trend in the legalization of cannabis for medicinal purposes in most US states, there is bound to be an even higher rise in the use of these substances. However, using an illicit substance for purposes restricted by law remains a crime [1,2]. Synthetic cannabinoids have been associated with transient coronary vasospasm, but very few cases have been associated with occlusive coronary artery disease [1,3]. In the case above, the patient had a total occlusion of his coronary artery in the absence of a personal or family history of heart disease, a few hours after synthetic cannabinoid use. 

Multiple theories have been proposed regarding the mechanism by which cannabis causes cardiovascular injury, but it remains unclear. Synthetic cannabinoids are known to bind with a higher affinity to the cannabinoid receptors CB1/CB2 compared to standard cannabis, but the direct mechanism regarding cardiovascular toxicity remains unexplained [2,4,5]. A few mechanisms have been proposed regarding cannabis and its toxic effect on the cardiovascular system; these include increased sympathetic activity, autonomic dysfunction, endothelial damage, platelet aggregation/factor VII activation, angiopathy, and higher carboxyhemoglobin levels [2].

Cannabis is also postulated to exert hemodynamic effects, which could initiate plaque rupture and promote thrombosis, contributing to a mismatch in oxygen demand and supply. In vitro, cannabis increased the expression of glycoproteins IIb-IIIa and P-selectin in a concentration-dependent manner, triggering platelet aggregation and factor VIII activation [6].

These mechanisms share a common factor: the decreased oxygen supply associated with increased oxygen demand [4-13]. Synthetic cannabinoids are full agonists at the cannabinoid receptors, which would explain their significant sympathomimetic effect [7]. Another mechanism proposed is increased platelet aggregation triggered by synthetic cannabinoids that strongly bind to the CB1/CB2 receptors, and this mechanism is especially relevant in this case, as the patient was found to have an acute thrombotic total occlusion [8,9].

The evaluation of patients suspected of synthetic cannabinoid-induced myocardial infarction is similar to that of patients suspected of having an acute coronary syndrome, as this remains a diagnosis of exclusion [10]. In these patients, we recommend complete cessation of cannabinoids as uninterrupted discontinuation of these substances has shown a decrease in their adverse effects over time [10]. Referral to addiction programs can also be offered to ensure appropriate follow-up and continuity of care.

Synthetic cannabinoids are not easily detected in urine drug screens because routine cannabinoid immunoassays do not cross-react with synthetic cannabinoids and their metabolites. This is a challenging concern as these substances emerge rapidly with new and different compounds, and this poses a challenge for immunoassay screening due to the time required to develop antibodies to the new metabolites [11,12].

Although not readily available, tests like tandem mass spectrometry, accurate mass technique, and simple method modification may be used when a urine drug screen is inconclusive due to its ability to target multiple metabolites in a single assay. However, its use in clinical practice is considerably limited [11,12].

## Conclusions

The purpose of this case report is to increase awareness and educate the public about the risks of synthetic cannabinoids due to their rising use among teenagers and young adults and the common misconception that they are safer alternatives to natural cannabis. These substances are chemically distinct and significantly more potent, acting as full agonists at CB1/CB2 receptors, which can lead to severe cardiovascular effects such as coronary vasospasm and even total artery occlusion, even in individuals without prior heart disease. The mechanisms of harm include increased sympathetic activity, endothelial damage, and platelet aggregation, contributing to decreased oxygen supply and increased demand. Compounding the danger, synthetic cannabinoids often go undetected in standard drug screenings, making diagnosis challenging. Therefore, public awareness and prevention are key to reducing their potentially life-threatening effects.
